# Does the Couse of Astragalus-Containing Chinese Herbal Prescriptions and Radiotherapy Benefit to Non-Small-Cell Lung Cancer Treatment: A Meta-Analysis of Randomized Trials

**DOI:** 10.1155/2013/426207

**Published:** 2013-12-18

**Authors:** Hailang He, Xianmei Zhou, Qian Wang, Yang Zhao

**Affiliations:** Department of Respiratory Medicine, Jiangsu Province Hospital of Chinese Medicine, Affiliated Hospital of Nanjing University of Chinese Medicine, 155 Hanzhong Road, Nanjing 210029, China

## Abstract

*Background.* Radiotherapy has been widely used for non-small-cell lung cancer (NSCLC), while its low efficacy and high toxicity raise big concerns. Astragalus (as a monarch drug)-containing Chinese herbal prescriptions and radiotherapy were frequently coused for NSCLC in China; however, the effects were not systematically analyzed. *Objective.* To evaluate the benefits of Astragalus-containing Chinese herbal prescriptions combined with radiotherapy for NSCLC. *Methods.* The randomized controlled trials involving NSCLC treatment with Astragalus-containing Chinese herbal prescriptions combined with radiotherapy were searched. The Review Manager 5.1 software was employed for data analysis. Funnel plot and Egger's test were applied to evaluate publication bias. *Results.* 29 eligible studies met our criteria. Of the studies, 8, 6, and 4 reported reduced risk of death at one year, two years, and three years, respectively. 26 studies revealed amended tumor response. Six studies showed improved Karnofsky performance status. Among the studies, 14 and 18 displayed a lowered white blood cells (WBC) toxicity and an ameliorated radiation pneumonia, respectively. *Conclusion.* Couse of Astragalus-containing Chinese herbal prescriptions and radiotherapy may benefit the patients with NSCLC via increasing the therapeutic effectiveness and reducing the toxicity of radiotherapy. To confirm the exact merits, further rigorously designed trials are warranted.

## 1. Introduction

It is estimated that 228,190 new cases of lung cancer are expected in 2013 and account for about 14% of cancer diagnoses [1]. As the leading cause of cancer death worldwide, lung cancer is guesstimated to occupy 26% of all female cancer deaths and 28% of all male cancer deaths in 2013 [2]. In China, the registered lung cancer mortality increased by 464.84% during the past 3 decades, and the crude mortality rates in 2008 were 47.51 per 100,000 men and 22.69 per 100,000 women [3]. Approximately eighty-five percent of all lung cancer occurrences are non-small-cell lung cancer (NSCLC) [4].

Radiotherapy, as a main therapeutic tool, has been widely used for NSCLC. It can be offered as adjunct for operable patients with resectable diseases, the primary local treatment for patients with medically or unresectable diseases, and an important palliative modality for patients with incurable diseases. Conventional radiotherapy has been used for the inoperable patients, but its outcomes were poor, with 5-year overall survival rates of only 15%–24% [5]. With the aid of advances in physics and computer technology, radiation delivery systems have been greatly improved, such as intensity-modulated radiation therapy, stereotactic radiation therapy, and particle therapy have been successfully used. Compared with previous methods, these new technologies can deliver even higher doses precisely to the tumor while minimizing doses to normal tissues, which leads to better tumor control with less toxicity [6]. Despite technological advances, radiotherapy for NSCLC still faces shortages of low efficacy and high toxicity; some patients even can not continue treatment due to serious adverse reactions.

In complementary and alternative medicines, Chinese herbal medicine has become increasingly popular for cancers patients undergoing radiotherapy or chemotherapy. A systematic review has showed that Chinese herbal medicine as an adjuvant therapy can reduce chemotherapy toxicity, prolong survival rate, enhance immediate tumor response, and improve Karnofsky performance score in advanced NSCLC patients [7]. Studies have indicated that traditional Chinese medicines have the potential to be effective systemic radiosensitizers that may be used to amplify radiation-induced toxicity on tumor tissues [8]. Furthermore, certain traditional Chinese medicine herbs may be used as radioprotectors that are able to ameliorate radiation-induced toxicity in normal tissues in cancer patients undergoing radiotherapy [9].

Particularly, the Astragalus-containing Chinese herbal prescriptions are frequently combined with radiotherapy for lung cancer in clinic. Astragali Radix (Astragalus, Chinese name, Huangqi) sourced from medicinal plants *Astragalus membranaceus* and *Astragalus membranaceus* var. *mongoholicus* (Leguminosae) has been listed in Chinese pharmacopoeia [10]. As an important herbal drug, Astragalus has been commonly used in Chinese traditional medicine for a long time, and also a variety of biological activities have been reported. Tin et al. have discovered that the total saponins obtained from Astragalus possessed significant antitumorigenic activity in HT-29 human colon cancer cells and tumor xenograft [11]. Study has shown that Astragalus exerted anticarcinogenic activity in colon cancer cells through the modulation of mTOR signaling and downregulation of COX-2, which together reduced VEGF level in tumor cells that could potentially suppress angiogenesis [12]. Cho and coworker have found that Astragalus root extracts could markedly enhance the tumoricidal activity of the peritoneal macrophages, act as a priming agent for the TNF production in tumor-bearing mice partially, restore the depressed immune functions in tumor-bearing mice in vivo, and induce the generation of cytotoxic cells against tumors in vitro [13]. What is more, it has been discovered that Astragalus root extracts also they could increase the IFN-*β* inducing activity of lactobacillus acidophilus in dendritic cells, which may exert immune-enhancing activity [14]. In clinical practice and most published trials, however, Astragalus usually combined with other herbal medicines as prescriptions, rarely as single-agent. Jinfukang, an oral liquid formulation, is one of the Astragalus-containing herbal preparations and has been approved by China Food and Drug Administration for the treatment of NSCLC. Meta-analysis has shown that Astragalus-based herbal formulas may increase effectiveness and reduce side effects of platinum-based chemotherapy when combined with chemotherapy [15].

There are a large number of published trials of Astragalus-containing Chinese herbal prescriptions, which are constituted in different forms, such as oral administration and intravenous injection, combined with radiotherapy for the treatment of NSCLC. However, the evidence for the effects of Astragalus-containing Chinese herbal prescriptions has not been systematically assessed. In the present study, a comprehensive systematic review is conducted on the efficacy of Astragalus-containing Chinese herbal prescriptions combined with radiotherapy for NSCLC.

## 2. Methods

### 2.1. Data Sources and Search Strategies

The literature searches were conducted in PubMed, EMbase, the Cochrane Central Register of Controlled Trials (CENTRAL) in the Cochrane Library, Chinese National Knowledge Infrastructure (CNKI), Wanfang Database (Wanfang), and Chinese Biomedical Literature Database (CBM). All of those searches ended on May 20, 2013.

The following terms were retrieved in databases as keywords or free-text terms: “non-small-cell lung cancer,” “radiotherapy,” “Chinese herbal medicine,” “*Astragalus membranaceus,*” and “randomized controlled trials” (and multiple synonyms for each term). The bibliographies of the included studies were searched for additional references. No restrictions were placed on the publication language. Two reviewers (Hailang He and Qian Wang) independently identified studies.

### 2.2. Inclusion Criteria

Studies included in the meta-analysis had to meet all of the following criteria. (1) Participants: NSCLC patients had to be diagnosed by pathological sections and were treated by radiotherapy. (2) Type of studies: only clinical randomized controlled trials (RCTs) were eligible. (3) Type of intervention: studies provided the treatment group with Astragalus-containing Chinese herbal prescriptions in combination with radiotherapy and the control group with radiotherapy alone were included for analysis. (4) Type of outcome measurements: overall survival rate, tumor response, and performance status were the main outcome measurements; other outcome measurements included reduction in the toxicity of radiotherapy, such as the inhibition of white blood cells and radiation pneumonitis, were also considered.

### 2.3. Data Extraction and Quality Assessment

The detailed method followed the reported one [16]. Two reviewers (Hailang He and Qian Wang) independently extracted data. The extracted data included authors, title of study, year of publication, study size, age and sex of the participants, details of methodological information, name and component of Chinese herbs, treatment process, details of the interventions, outcomes, and adverse effects for each study. Any disagreements were resolved by consensus or by a third reviewer (Xianmei Zhou).

Methodological quality of RCTs was assessed independently by two review authors (Hailang He and Qian Wang) with the criteria in the Cochrane Handbook for Systematic Reviews of Interventions 5.1.0 [17]. Sequence generation, allocation concealment, blinding (or masking), incomplete data assessment, selective outcome reporting, and other sources of bias were assessed with three potential responses: yes, no, and unclear. Disagreements between review authors were resolved by discussion or with the third author (Xianmei Zhou).

### 2.4. Outcome Measures

The risk ratios of death at one, two, and three years were calculated as the proportion that died in the Astragalus-containing Chinese herbal prescriptions plus radiotherapy group divided by this proportion in the radiotherapy alone group. Tumor response was calculated as the number of patients with complete response (CR) plus partial response (PR) based on the WHO scale [18] divided by the total number of patients in each treatment group. The performance status of patients was investigated based on the Karnofsky performance score (KPS) [19], and the improved performance status was calculated as the number of patients with improved performance status (>10-point increase) divided by the total. Radiotherapy toxicity was investigated based on the WHO scale [18]; the reduction of radiotherapy toxicity was calculated as the number of patients with any toxicity (WHO grades 1 2 3 4) divided by the total number of patients in each treatment group (WHO grades 0 1 2 3 4).

### 2.5. Data Analysis

The Review Manager 5.1 software (http://www.cochrane.org/) was employed for data analysis. The effect data is expressed as relative risk (RR) with 95% confidence interval (CI). If the heterogeneity exists in pooled studies (*I* ^2^ > 50%), a random model was applied; otherwise, the fix model was applied. Statistical significant difference was considered as *P* < 0.05. Funnel plot was applied to evaluate the potential publication bias if at least ten trials were available for a meta-analysis [17]. Egger's test was further conducted to evaluate funnel plot asymmetry with STATA 12.1 software.

## 3. Results

### 3.1. Description of Studies

**Figure 1:**
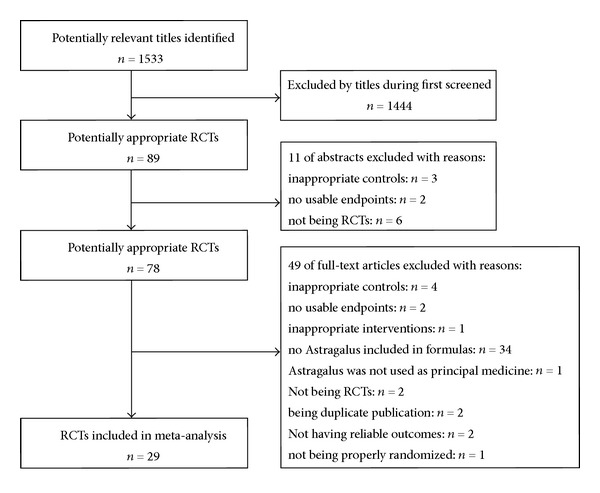
Flow diagram showing the trial selection process for the systematic review.

After primary search of titles and abstracts from the 6 databases, 1533 trials were screened out from electronic and manual searches as shown in Figure 1, of which 89 were identified as requiring relevant abstracts retrieval. Close reviewing of the 89 abstracts excluded 11 because of inappropriate controls (*n* = 3), no usable endpoints (*n* = 2), and not being RCTs (*n* = 6). 78 Full-text articles were further reviewed for eligibility. 49 Articles were excluded due to inappropriate controls (*n* = 4), having no usable endpoints (*n* = 2), inappropriate interventions (*n* = 1), having no Astragalus included in formulas (*n* = 34), having no Astragalus used as principal medicine (*n* = 1), not being RCTs (*n* = 2), being duplicate publication (*n* = 2), not having reliable outcomes (*n* = 2), and not properly randomized (*n* = 1). Thus, the total of 29 eligible studies were accepted for the current meta-analysis.

A total of 2547 participants were involved in the 29 studies, at which 1,298 patients participated in radiotherapy combined with Astragalus-containing Chinese herbal prescriptions (1 patients dropped out) and 1,241 in radiotherapy alone (7 patients dropped out). All of these studies were conducted in China. The baseline including age, gender, histopathology, and TNM stage of all studies was comparable.

### 3.2. Characteristics of the Eligible Studies

Table 1 shows characteristics of the eligible studies. As shown, all of the studies were conducted in China and published between 2002 and 2013 in Chinese journals. The stages of NSCLC TNM of the patients recruited in the current studies were as follows: 5 studies [20–24] were at I to IV but mentioned ambiguously; 4 [25–28], 1 [29], 5 [30–34], and 12 [35–46] studies were at II to III, II to IV, III, and III to IV, respectively. The other 2 studies [47, 48] did not mention the stage condition. 18 Studies [20, 25–31, 34–38, 42, 44, 47, 48] used the conventional radiotherapy; three-dimensional conformal radiotherapy was applied in 8 studies [21–24, 32, 41, 43, 46]; 2 studies [39, 45] employed conventional radiotherapy combined with three-dimensional conformal radiotherapy; and 1 study [33] adopted stereotactic radiation therapy. The dose of radiation therapy varied from 45 to 70 Gy in the included studies. Of all Astragalus-containing Chinese herbal prescriptions, the oral Astragalus prescriptions were used in 12 studies [20, 23, 24, 29–33, 35, 42, 44, 47] and Aidi injection [21, 25–28, 34, 38], Kangai Injection [36, 37, 46], Shenqifuzheng injection [39, 41, 43, 45] and Delisheng injection [40] were used in 7, 3, 4, and 1 studies, respectively. Astragalus polysaccharide injection was involved in 1 study [22] and 1 study [48] took Zhenqifuzheng capsules. The durations of the treatments varied from 3 to 9 weeks in the included studies.

### 3.3. Risk of Bias in Included Studies

The risk of bias of each study was assessed by the Cochrane Handbook for Systematic Reviews of Interventions 5.1.0. Of all the involved studies that claimed randomization, only 5 provided the specific information on the randomization method. None used a central randomization procedure to ensure concealment of treatment allocation. No trials mentioned the blinding procedures. Of all the trials, only 5 mentioned drop-out data, of which 3 considered the patients who dropped out of the study as the treatment failure (death). This is similar to an intention-to-treat analysis [49]. In general, all of 29 RCTs have an unclear risk of bias.

### 3.4. Outcome Measures

#### 3.4.1. Survival Status

**Figure 2:**
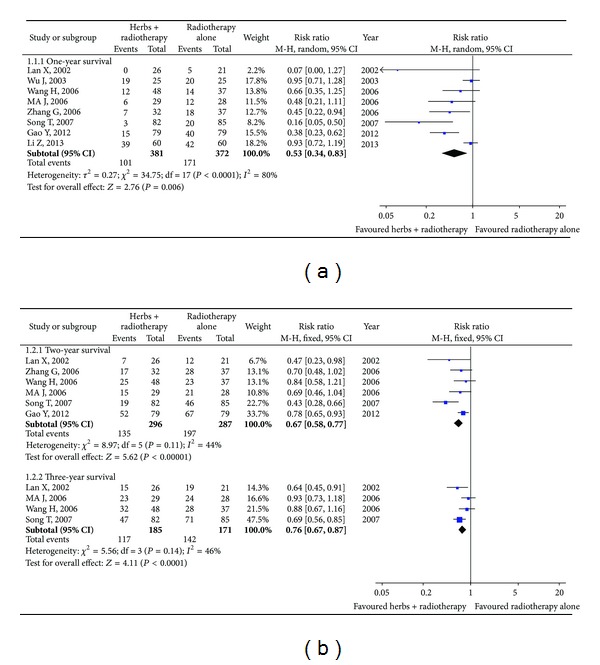
One-year, two-year, and three-year survivals with Astragalus-containing Chinese herbal prescriptions and radiotherapy versus radiotherapy alone.

As shown in Figure 2, 8 studies [20, 23, 25, 31, 32, 34, 35, 37] including 753 patients observed the one-year survival. As the 8 trials did not show homogeneity (chi-square = 34.75, *I* ^2^ = 80%, *P* < 0.0001), the random-effects model was used for statistical analysis. The combined effects showed that the patients receiving Astragalus-containing Chinese herbal prescriptions plus radiation therapy had significantly lower risk of death at one year when compared with the radiotherapy alone group (RR 0.53; 95% CI, 0.34 to 0.83).

Six trials (Figure 2) including 583 patients exhibited the two-year survival [20, 23, 31, 32, 35, 37]. Due to the homogeneity of the trials (chi-square = 8.97, *I* ^2^ = 44%, *P* = 0.11), fixed-effects model was used for the analysis. The results revealed that the patients with the combination treatment of Astragalus-containing Chinese herbal prescriptions and radiation therapy showed significantly lower risk of death at two years compared with the radiotherapy alone group (RR 0.67; 95% CI, 0.58 to 0.77). 

The same result was also observed in 4 studies [20, 31, 35, 37] including 356 patients (Figure 2, fixed-effects model was used). The combination treatment displayed significantly lower risk of death at three years (RR 0.76; 95% CI, 0.67 to 0.87).

Due to the small number of studies in the survival status analysis, funnel plots were not used to assess the risk of publication bias.

#### 3.4.2. Tumor Response

**Figure 3:**
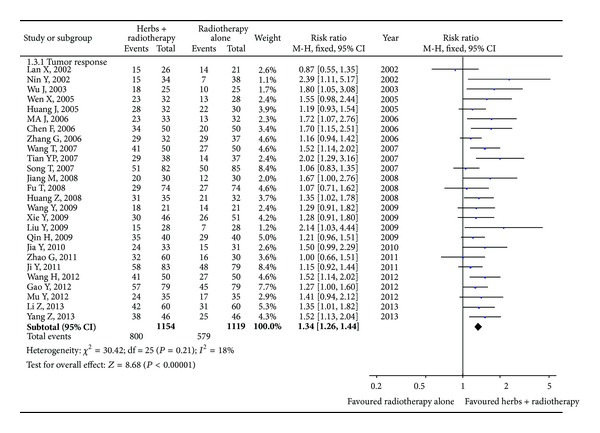
Tumor response with Astragalus-containing Chinese herbal prescriptions and radiotherapy versus radiotherapy alone.

**Figure 4:**
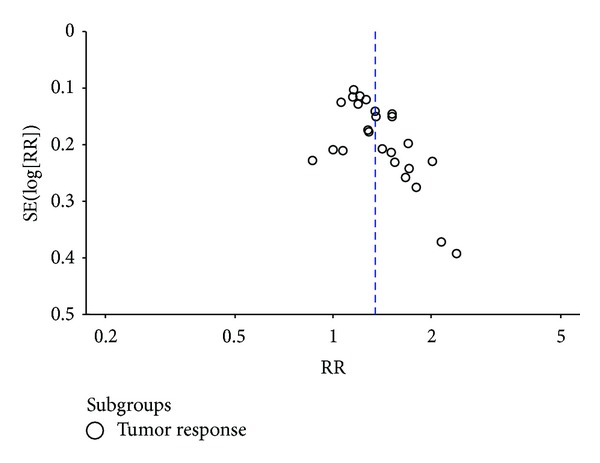
Funnel plot of studies testing for tumor response.

26 Studies [20–23, 25–29, 32–48] including 2,273 patients that reported the tumor response were identified (Figure 3). The analytical results with fixed-effects model (homogeneity, chi-square = 30.42, *I* ^2^ = 18%, *P* = 0.21) demonstrated that the combination treatment of Astragalus-containing Chinese herbal prescriptions and radiation therapy was associated with a significant increase in the number of patients reported complete and partial response when compared with the radiotherapy alone group (RR 1.34; 95% CI, 1.26 to 1.44). The symmetry of the funnel plot was not clear (Figure 4). Egger's test indicated that the effect of publication bias was significant (*t* = 3.35, 95% CI, 0.74 to 3.11, *P* = 0.003).

#### 3.4.3. Performance Status

**Figure 5:**
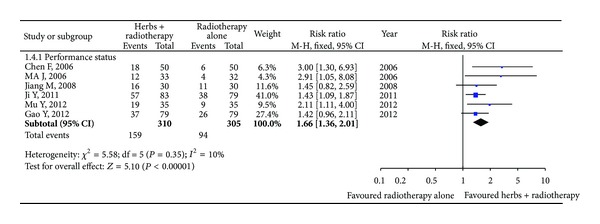
Improved Karnofsky performance status with Astragalus-containing Chinese herbal prescriptions and radiotherapy versus radiotherapy alone.

As can be seen in Figure 5, 6 studies [23, 36, 37, 40, 44, 45] including 615 patients that reported the performance status about the improvement of KPS (ten-point cutoff) were involved. The 6 trials showed homogeneity (chi-square = 5.58, *I* ^2^ = 10%, *P* = 0.35), and fixed-effects model was used. The combination treatment with Astragalus-containing Chinese herbal prescriptions plus radiation therapy significantly improved the performance status when compared with the radiotherapy alone group (RR 1.66; 95% CI, 1.36 to 2.01). The number of studies reporting performance status was less than ten, so a funnel plot was not applicable.

#### 3.4.4. Reduction in Radiotherapy Toxicity

**Figure 6:**
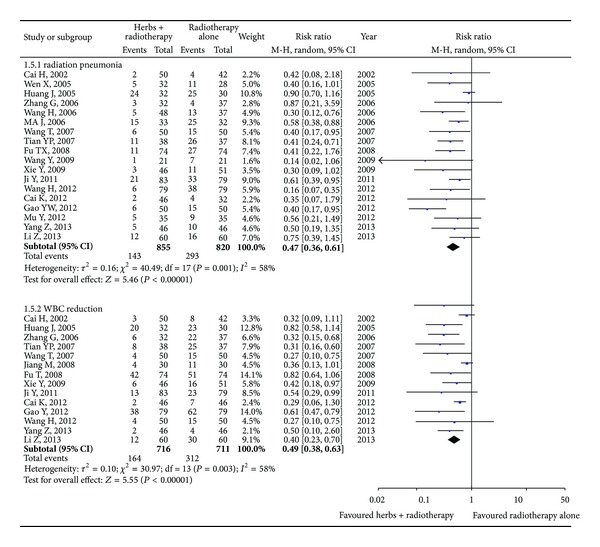
Radiation pneumonia and WBC reduction with Astragalus-containing Chinese herbal prescriptions and radiotherapy versus radiotherapy alone.

**Figure 7:**
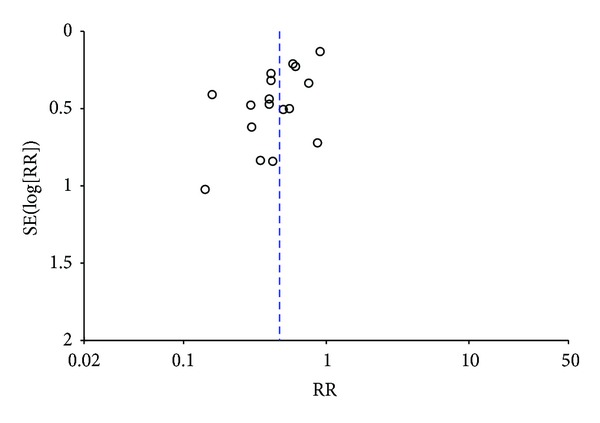
Funnel plot of studies testing for radiation pneumonia.

The radiation pneumonia is one of the main side effects that radiation therapy resulted in. We identified 18 studies [23, 24, 26, 28–32, 34, 37, 38, 41, 43–46, 48] including 1,675 patients with the radiation pneumonia (Figure 6). The statistical analysis with random-effects model revealed that Astragalus-containing Chinese herbal prescriptions plus radiation therapy had a significant decrement in radiation pneumonia when compared with the radiotherapy alone group (RR 0.47; 95% CI, 0.36 to 0.61). The funnel plot revealed an asymmetrical distribution of studies around the line of identity, indicating the possibility of publication bias (Figure 7). Egger's test showed that the effect of publication bias was significant (*t* = −3.74, 95% CI, −2.83 to −0.78, *P* = 0.002).

**Figure 8:**
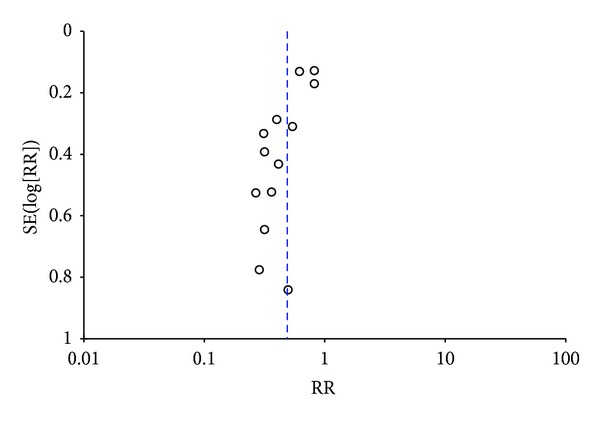
Funnel plot of studies testing for WBC reduction.

As mentioned above, the toxicity is a severe problem that radiotherapy is facing. As can be seen in Figure 6, 14 studies [23, 24, 28–30, 32, 34, 38–41, 44, 46, 48] including 1,427 patients reported the WBC toxicity. The results demonstrated that Astragalus-containing Chinese herbal prescriptions plus radiation therapy possessed a significant reduction in WBC toxicity when compared with the radiotherapy alone group (RR 0.49; 95% CI, 0.38 to 0.63). The funnel plot revealed an asymmetrical distribution of studies around the line of identity, indicating the possibility of publication bias (Figure 8). Egger's test indicated that the effect of publication bias was significant (*t* = −4.66, 95% CI, −2.93 to −1.06, *P* = 0.001).

## 4. Discussion

In the present meta-analysis, 29 studies with 2,547 individuals suffering from NSCLC were selected out. The main findings revealed that combining radiotherapy with Astragalus-containing Chinese herbal prescriptions in the treatment of NSCLC may increase survival, tumor response, and performance status and reduce radiotherapy toxicity when compared with the radiotherapy alone. However, due to the generally weak methodological quality of the currently included studies, we are unable to make solid conclusions, and confirmation must await investigation in future trials.

The methodological quality and report of the majority of trials were variable and often inadequate. Of 29 trials, only 5 provided information on the randomization method and none reported a central randomization procedure to ensure concealment of treatment allocation. Blinded assessors were not mentioned in any study. To analyze survival time, it is essential that standards in the quality of the studies require the authors to specify how they handle patients who are lost to followup, the percentage of patients lose to followup, and whether those patients are monitored in the analysis [50]. However, only 3 reported considering the patients who were lose to followup as they had died; none of the other studies provided this information in current meta-analysis. The lack of information in many reports may not necessarily indicate poor implementation within the trial, but without this information, the level of bias within each trial is difficult to assess. In addition, there was between-study heterogeneity in the evidence for improved survival at one year, as well as in the evidence for reduced radiotherapy toxicity. Moreover, all studies included in this systematic review used an “A + B versus B” design where patients were randomized to receive Chinese herbal prescriptions plus radiotherapy versus radiotherapy alone, without a rigorous control for placebo effect. This kind of design is likely to generate false positive results [51]. What is more, although the radiation technology was the same between the groups of Chinese herbal prescriptions plus radiotherapy and the radiotherapy alone in every eligible trail, of all the trails included in this meta-analysis, some used the conventional radiotherapy, some used three-dimensional conformal radiotherapy, and one adopted stereotactic radiation therapy. Hence, performance bias could be caused. Besides, funnel plots and Egger's test showed that there was evidence of publication bias which was another limitation of the present meta-analysis.

Astragalus possesses an important position in traditional Chinese medicine system. It has been used for almost all the disease caused by “qi deficiency” (life energy), which has been associated with cellular immune dysfunction [52]. The main constituents of Astragalus are triterpene saponins, flavonoids, and Astragalus polysaccharides [53]. Among the constituents, especially, the astragalus polysaccharide integrated with vinorelbine and cisplatin (VC) showed a significantly improved quality of life in patients with advanced NSCLC compared with VC alone [54]. Furthermore, as an immunomodulator [55], the astragalus polysaccharides enhances the immune responses [56] and resists the immunosuppression [57]. As a key ingredient of the current herbal prescriptions, those effects might directly associate with the benefits to the patients with NSCLC when coused with radiotherapy. However, to clarify the herbal prescriptions function as an adjunct to radiotherapy, future studies focused on the specific mechanisms and bioactive components of herbal prescriptions themselves are essential. What is more, improvement in the methodological quality of randomized controlled trials is critical for future research and more methodologically rigorous studies are justified to confirm or refute the effects reported here. Besides, future trials need to ensure that the reporting follows the CONSORT guidelines [58].

## 5. Conclusion

In conclusion, we found evidence that Astragalus-containing Chinese herbal prescriptions may increase effectiveness and reduce the toxicity of radiotherapy when combined with radiotherapy. To confirm the exact merit, further rigorously controlled trials are warranted.

## Figures and Tables

**Table 1:** Characteristics of the eligible studies.

Study	No.	Stage	Protocol*	Herbal ingredients	Dose of RT	Duration (week)
Cai et al., 2002 [30]	92	III	CRT + AP	Astragalus, Codonopsis Radix, Poria, Alismatis Rhizoma, Ophiopogonis Radix, Schisandra Chinensis Fructus, Eriobotryae Folium, Rehmanniae Radix Praeparata, Corni Fructus, Cyperi Rhizoma, and Glycyrrhizae Radix et Rhizome.	60–70 Gy	6-7
Nin et al., 2002 [47]	72	NR	CRT + AP	Ginseng Radix et Rhizoma, Atractylodis Macrocephalae Rhizoma, Astragalus, Poria, Lycii Fructus, Lilii Bulbus, Coicis Semen, Armeniacae Semen Amarum, and Chuanxiong Rhizoma.	55–65 Gy (2 Gy/f)	5–7
Lan and Jiang, 2002 [35]	47	III-IV	CRT + AP	Manis Squama, Glehniae Radix, Corni Fructus, Astragalus, Fritillariae Cirrhosae Bulbus, Ophiopogonis Radix, Polygoni Multiflori Radix, Rehmanniae Radix Preparata, Dioscoreae Rhizoma, Alismatis Rhizoma, and Glycyrrhizae Radix et Rhizome.	60 Gy	6
Wu, 2003 [25]	50	IIb-III	CRT + ADI	Ginseng Radix et Rhizoma, Astragalus, Mylabris, and Acanthopanacis senticosi Radix et Rhizoma Seu Caulis.	60–70 Gy (2 Gy/f)	6-7
Wen, 2005 [26]	64	II-III	CRT + ADI	Ginseng Radix et Rhizoma, Astragalus, Mylabris, and Acanthopanacis senticosi Radix et Rhizoma Seu Caulis.	60–70 Gy (1.8–2.0 Gy/f)	6-7
Huang et al., 2005 [29]	62	II–IV	CRT + AP	Astragalus, Atractylodis Macrocephalae Rhizoma, Pseudostellariae Radix, Lycii Fructus, Spatholobi Caulis, Carthami Flos, Sappan Lignum, Lonicerae Japonicae Flos, Galli Gigerii Endothelium Corneum, Dendrobii Caulis, Glehniae Radix, and Poria.	61–66 Gy	NR
Wang et al., 2006 [31]	85	III	CRT + AP	Astragalus, Pseudostellariae Radix, Angelicae Sinensis Radix, Scutellariae Barbatae Herba, Coicis Semen, Glycyrrhizae Radix et Rhizoma, Paeoniae Radix Alba, Rehmanniae Radix Preparata, Lilii Bulbus, Ophiopogonis Radix, Scrophulariae Radix, Fritillariae Cirrhosae Bulbus, and Platycodonis Radix.	70 Gy (2 Gy/f)	7
Chen and Wang, 2006 [36]	100	III-IV	CRT + KAI	Ginseng Radix et Rhizoma, Astragalus, and Sophorae Flavescentis Radix.	65–70 Gy (2 Gy/f)	6.5–7
Ma, 2006 [37]	69	III-IV	CRT + KAI	Ginseng Radix et Rhizoma, Astragalus, and Sophorae Flavescentis Radix.	65~70 Gy (2 Gy/f)	6.5–7
Zhang et al., 2006 [32]	69	III	3D-CRT + AP	Pseudostellariae Radix, Glehniae Radix, Coicis Semen, Ophiopogonis Radix, Asparagi Radix, Astragalus, Lycii Fructus, Angelicae Sinensis Radix, Schisandra Chinensis Fructus, Schisandra Chinensis Fructus, Hedyotis Herba, Glycyrrhizae Radix et Rhizoma, Lonicerae Japonicae Flos, Atractylodis Macrocephalae Rhizoma, Moutan Cortex, Rehmanniae Radix Praeparata, Codonopsis Radix, and Poria.	56–62 Gy (4-5 Gy/f)	3–5
Tian and Wang, 2007 [38]	75	III-IV	CRT + ADI	Ginseng Radix et Rhizoma, Astragalus, Mylabris, and Acanthopanacis Senticosi Radix et Rhizoma Seu Caulis.	60 Gy (1.8–2.0 Gy/f)	6
Wang et al., 2007 [39]	100	III-IV	CRT & 3D-CRT + SQFZI	Codonopsis Radix and Astragalus.	60–70 Gy (2 Gy/f)	6-7
Song et al., 2007 [20]	167	I–IV	CRT + AP	Manis Squama, Glehniae Radix, Alismatis Rhizoma, Fritillariae Cirrhosae Bulbus, Astragalus, Corni Fructus, Ophiopogonis Radix, Polygoni Multiflori Radix, Scrophulariae Radix, Rehmanniae Radix Praeparata, Glycyrrhizae Radix et Rhizoma, and Dioscoreae Rhizome.	60–70 Gy (2 Gy/f)	6-7
Jiang and Xu, 2008 [40]	60	III-IV	CRT + DLSI	Ginseng Radix et Rhizoma, Astragalus, Mylabris, and Bufonis Venenum.	60–70 Gy (1.8–2.0 Gy/f)	6-7
Fu et al., 2008 [48]	148	NR	CRT + ZQFZC	Astragalus and Angelicae sinensis Radix.	60–70 Gy (2 Gy/f)	6-7
Huang and Hou, 2008 [21]	67	I–IV	3D-CRT + ADI	Ginseng Radix et Rhizoma, Astragalus, Mylabris, and Acanthopanacis senticosi Radix et Rhizoma Seu Caulis.	45–55 Gy (5-6 Gy/f)	3-4
Xie et al., 2009 [41]	97	III-IV	3D-CRT + SQFZI	Codonopsis Radix and Astragalus.	60–70 Gy	6-7
Liu et al., 2009 [42]	56	III-IV	CRT + AP	Ganoderma, Hedyotis Herba, Chuanxiong Rhizoma, Stephaniae Tetrandrae Radix, and Astragalus.	64–88 Gy (2 Gy/f)	6–9
Wang et al., 2009 [43]	42	III-IV	3D-CRT + SQFZI	Codonopsis Radix and Astragalus.	70 Gy (2 Gy/f)	7
Qin et al., 2009 [22]	80	I–IV	3D-CRT + APS	Astragalus Polysaccharide.	50–70 Gy (2 Gy/f)	5–7
Jia et al., 2010 [27]	64	IIb-III	CRT + ADI	Ginseng Radix et Rhizoma, Astragalus, Mylabris, and Acanthopanacis Senticosi Radix et Rhizoma Seu Caulis.	60–70 Gy (2 Gy/f)	NR
Ji et al., 2011 [44]	162	III-IV	CRT + AP	Codonopsis Radix, Chuanxiong Rhizoma, Atractylodis Macrocephalae Rhizoma, Astragalus, and Rehmanniae Radix Praeparata.	60–70 Gy (2 Gy/f)	6
Zhao et al., 2011 [33]	90	III	SBRT + AP	Astragalus, Atractylodis Macrocephalae Rhizoma, Poria, Rehmanniae Radix, Chuanxiong Rhizoma, Ginseng Radix et Rhizoma, Angelicae Sinensis Radix, Glycyrrhizae Radix et Rhizoma, and Paeoniae Radix Alba.	60–70 Gy (2-3 Gy/f)	5-6
Gao et al., 2012 [23]	158	I–IV	3D-CRT + AP	Ginseng Radix et Rhizoma, Astragalus, Angelicae sinensis Radix, Gastrodiae Rhizoma, Rehmanniae Radix Preparata, Alismatis Rhizoma, Cassiae Semen, Cervi Cornu, Asari Radix et Rhizoma, Lycii Fructus, and Cuscutae Semen.	60–66 Gy (1.8 Gy/f)	6–6.6
Mu et al., 2012 [45]	70	III-IV	CRT & 3D-CRT + SQFZI	Codonopsis Radix and Astragalus.	60–70 Gy	6-7
Wang et al., 2012 [46]	100	III-IV	3D-CRT + KAI	Ginseng Radix et Rhizoma, Astragalus, and Sophorae Flavescentis Radix.	60–70 Gy (2 Gy/fr)	6-7
Cai et al., 2012 [24]	89	I–IV	3D-CRT + AP	Astragalus, Pseudostellariae Radix, Hedyotis Herba, Schisandra Chinensis fructus, Ophiopogonis Radix, Mori Cortex, Armeniacae Semen Amarum, Pinelliae Rhizoma, Trichosanthis Pericarpium, Curcumae Radix, Eriobotryae Folium, and Citri Reticulatae Pericarpium.	60–70 Gy	6-7
Li et al., 2013 [34]	120	III	CRT + ADI	Ginseng Radix et Rhizoma, Astragalus, Mylabris, and Acanthopanacis Senticosi Radix et Rhizoma Seu Caulis.	60–70 Gy (2 Gy/f)	6-7
Yang, 2013 [28]	92	II-III	CRT + ADI	Ginseng Radix et Rhizoma, Astragalus, Mylabris, and Acanthopanacis Senticosi Radix et Rhizoma Seu Caulis.	60–70 Gy (2 Gy/f)	6-7

Abbreviations—No.: number of participants; RT: radiotherapy; AP: Astragalus prescription; CRT: conventional radiotherapy; 3D-CRT: three-dimensional conformal radiotherapy; SBRT: stereotactic radiation therapy; ADI: Aidi injection; KAI: Kangai injection; SQFZI: Shenqi fuzheng injection; DLSI: Delisheng injection; ZQFZC: Zhenqifuzheng capsules; APS: Astragalus polysaccharide; f: fraction; NR: not reported.

*Treatment group intervention.

## References

[1] Siegel R, Naishadham D, Jemal A (2013). Cancer statistics, 2013. *A Cancer Journal for Clinicians*.

[2] Keith RL, Miller YE (2013). Lung cancer chemoprevention: current status and future prospects. *Nature Reviews*.

[3] She J, Yang P, Hong QY, Bai CX (2013). Lung cancer in China: challenges and interventions. *Chest*.

[4] Yang Y, Li H, Hou SC, Hu B, Liu J, Wang J (2013). The noncoding RNA expression profile and the effect of lncRNA AK126698 on cisplatin resistance in Non-Small-Cell Lung Cancer Cell. *PLoS ONE*.

[5] Grutters JPC, Kessels AGH, Pijls-Johannesma M, de Ruysscher D, Joore MA, Lambin P (2010). Comparison of the effectiveness of radiotherapy with photons, protons and carbon-ions for non-small cell lung cancer: a meta-analysis. *Radiotherapy and Oncology*.

[6] Ishikura S (2012). Optimal radiotherapy for non-small-cell lung cancer: current progress and future challenges. *General Thoracic and Cardiovascular Surgery*.

[7] Li SG, Chen HY, Ou-Yang CS (2013). The efficacy of Chinese herbal medicine as an adjunctive therapy for advanced non-small cell lung cancer: a systematic review and meta-analysis. *PLoS ONE*.

[8] Jia LL, Ma SM, Hou X (2013). The synergistic effects of traditional Chinese herbs and radiotherapy for cancer treatment. *Oncology Letters*.

[9] Lee T-K, Johnke RM, Allison RR, O’Brien KF, Dobbs LJ (2005). Radioprotective potential of ginseng. *Mutagenesis*.

[10] National Pharmacopoeia Committee (2010). *Pharmacopoeia of People’s Republic of China*.

[11] Tin MMY, Cho C-H, Chan K, James AE, Ko JKS (2007). Astragalus saponins induce growth inhibition and apoptosis in human colon cancer cells and tumor xenograft. *Carcinogenesis*.

[12] Law PC, Auyeung KK, Chan LY, Ko JK (2012). Astragalus saponins downregulate vascular endothelial growth factor under cobalt chloride-stimulated hypoxia in colon cancer cells. *BMC Complementary and Alternative Medicine*.

[13] Cho WCS, Leung KN (2007). In vitro and in vivo anti-tumor effects of *Astragalus membranaceus*. *Cancer Letters*.

[14] Frøkiær H, Henningsen L, Metzdorff SB (2012). Astragalus root and elderberry fruit extracts enhance the IFN-*β* stimulatory effects of Lactobacillus acidophilus in murine-derived dendritic cells. *PLoS ONE*.

[15] McCulloch M, See C, Shu X-J (2006). Astragalus-based Chinese herbs and platinum-based chemotherapy for advanced non-small-cell lung cancer: meta-analysis of randomized trials. *Journal of Clinical Oncology*.

[16] Wu L, Chen YB, Xu YJ (2013). Oral huangqi formulae for stable chronic obstructive pulmonary disease: a systematic review and meta-analysis. *Evidence-Based Complementary and Alternative Medicine*.

[17] Higgins JPT, Green S Cochrane Handbook for Systematic Reviews of Interventions Version 5.1.0. http://www.cochrane.org/training/cochrane-handbook.

[18] The World Health Organization (1979). *WHO Handbook for Reporting Results of Cancer Treatment*.

[19] Yates JW, Chalmer B, McKegney FP (1980). Evaluation of patients with advanced cancer using the Karnofsky performance status. *Cancer*.

[20] Song TT, Jiang YH, Lan XZ (2007). The observation of the rapeutic efficacy of Chinese medicine combined with radiotherapy in treatment of elderly patients with NSCLC. *Journal of Practical Oncology*.

[21] Huang ZG, Hou Y (2008). An evaluation of the efficacy of AD Injection in combination with three-dimensional conformal radiotherapy for elderly patients with non-small-cell-lung carcinoma. *Journal of YouJiang Medical College for Nationalities*.

[22] Qin H, Niu D, Jiang C (2009). Effect of astragalus polysaccharide in combination with three-dimensional conformal radiotherapy on elderly patients with non-small cell lung cancer. *Chinese Journal of Clinical Oncology*.

[23] Gao YW, Yin LJ, Ding TG, Wang J, Chen W (2012). Shenqi ten blindly particles combined with radiotherapy for elderly patients with non-small cell lung cancer. *Chinese Journal of Clinical Oncology and Rehabilitation*.

[24] Cai K, Liu JB, Huang CJ, Yu ZS, Liao TH, Huang LL (2012). Effects of YiQi YangYin drugs on life quality and immunologic function in senile patients with local advanced non-small-cell lung cancer undergoing three dimensional conformal radiotherapy. *Western Journal of Traditional Chinese Medicine*.

[25] Wu J (2003). Observation of effects of Aidi Injection in the radiotherapy of non-small cell lung cancer. *China Journal of Cancer Prevention and Treatment*.

[26] Wen XX (2005). Clinical observation of the effects of radiotherapy in combination with Aidi Injection in treatment of elderly patients with non-small cell lung cancer. *Chinese Journal of Hospital Pharmacy*.

[27] Jia YS, Lin BH, Wu SQ (2010). Clinical research on Aidi Injection combined with radiotherapy for non-small-cell lung cancer. *Chinese Archives of Traditional Chinese Medicine*.

[28] Yang Z (2013). Clinical study on AiDi Injection combined with radiotherapy for 46 cases of non-small cell lung cancer. *China Health Industry*.

[29] Huang J, Li PW, Jia LQ (2005). Complementary effect of Fuzheng Zengxiao Fomular combining with radiotherapy in non-small cell lung cancer. *Journal of China-Japan Friendship Hospital*.

[30] Cai H-B, Dai F-G, Min Q-F, Shi M, Miao J-X, Luo R-C (2002). Clinical study of the effects of radiotherapy in combination with traditional Chinese medicine on non-small cell lung cancer. *Journal of First Military Medical University*.

[31] Wang HJ, Wang ZX, Guo JF (2006). The clinical study on Jiawei Baihe Gujin decoction combined with radiotherapy in the treatment of stage III non-small cell lung cancer. *Chinese Archives of Traditional Chinese Medicine*.

[32] Zhang GM, Xiao BR, Mao RK, Long HL, Zhao XM (2006). Study on NSCLC treated with Chinese medicine combined with three-dimensional conformal radiotherapy. *Chinese Journal of Clinical Oncology and Rehabilitation*.

[33] Zhao GY, Dong W, Feng JJ (2011). The observation of the rapeutic efficacy of Chinesse medicine combined with radiotherapy in treatment of elderly patients with stage IIIa/IIIb non-small cell lung cancer. *Cancer Research and Clinic*.

[34] Li ZY, Nin SQ, Yi TN, Liu GQ (2013). The observation of the action-enhancing and toxicity-reducing efficacy of AiDi Injection combined with radiotherapy in treating lung cancer. *Journal of Clinical Pulmonary Medicine*.

[35] Lan XZ, Jiang YH (2002). Observation on therapeutic effects of 26 cases of senile late non-small cell pulmonary carcinoma treated with radiotherapy combined with chinese drugs. *Journal of Traditional Chinese Medicine*.

[36] Chen F, Wang XL (2006). The observation of the action-enhancing and toxicity-reducing efficacy of KangAi Injection combined with radiotherapy in treating Stage III/IV non-small cell lung cancer. *China Journal of Chinese Materia Medica*.

[37] Ma JQ (2006). The observation of the synergism effects of KangAi Injection combined with radiotherapy in treating advanved non-small-cell lung cancer. *China Journal of Chinese Materia Medica*.

[38] Tian YP, Wang QC (2007). The observation of the rapeutic efficacy of AiDi Injection combined with radiotherapy in treatment of elderly patients with advanced NSCLC. *Pharmacology and Clinics of Chinese Materia Medica*.

[39] Wang TJ, Jiang Y, Wang HY, Zhao ZF (2007). The observation of the rapeutic efficacy of Shenqifuzheng Injection combined with radiotherapy in treating limited and advanced non-small cell lung cancer. *Modern Oncology*.

[40] Jiang M, Xu JH (2008). The clinical study on Delisheng (DLS) combined with radiotherapy in the treatment of advanced non small cell lung cancer (NSCLC). *Guide of China Medicine*.

[41] Xie YS, Zhang Q, Wang R (2009). Shenqifuzheng Injection combined with 3-dimensional conformal radiotherapy for 46 cases of advanced non-small cell lung cancer. *Herald of Medicine*.

[42] Liu Y, Jiang HP, Wang YL (2009). The clinical study of treating advanced non-small cell lung cancer combining radiotherapy with Taile oral liquid. *Journal of Bethune Military Medical College*.

[43] Wang YX, Zheng SM, Fan HM, He R, Li J, Xiao XR (2009). Shenqi Fuzheng Injection combined with three dimensional conformal radiotherapy for old patient's advanced non-small cell lung cancer. *Chongqing Medicine*.

[44] Ji YH, Zhang JL, Ni MX, Cai J, Liu JB (2011). Effects of Bukangling on the radiotherapy for advanced non-small-cell lung cancer. *Journal of Chinese Practical Diagnosis and Therapy*.

[45] Mu Y, Ruan YM, Zhou J (2012). Clinical study on Shenqifuzheng Injection combined with radiotherapy for 35 cases of advanced non-small cell lung cancer. *Guiding Journal of Traditional Chinese Medicine and Pharmacy*.

[46] Wang HY, Jia XJ, Chen YB, Wang TJ (2012). The observation of the rapeutic efficacy of KangAi Injection combined with three dimensional conformal radiotherapy in treating locally anvanced non-small cell lung cancer. *Chinese Journal of Gerontology*.

[47] Nin YF, Yang LZ, Yang BL, Li YH (2002). Effects of Jiawei Shiquan Fomular on immunologic function in patients with non-small-cell lung cancer undergoing radiotherapy. *Chinese Journal of Coal Industry Medicine*.

[48] Fu TX, Tang Q, Nie B (2008). Clinical observation of Zhenqi Fuzheng capsules combined by radiotherapy in treatment of elderly patients with advanced NSCLC. *Chinese Journal of Pharmacoepidemiology*.

[49] Heritier SR, Gebski VJ, Keech AC (2003). Inclusion of patients in clinical trial analysis: the intention-to-treat principle. *Medical Journal of Australia*.

[50] Hewitt C, Hahn S, Torgerson DJ, Watson J, Bland JM (2005). Adequacy and reporting of allocation concealment: review of recent trials published in four general medical journals. *British Medical Journal*.

[51] Ernst E, Lee M (2008). A trial design that generates only “positive” results. *Journal of Postgraduate Medicine*.

[52] Block KI, Mead MN (2003). Immune system effects of Echinacea, Ginseng, and Astragalus: a review. *Integrative Cancer Therapies*.

[53] Chu C, Qi L-W, Liu E-H, Li B, Gao W, Li P (2010). Radix astragali (Astragalus): latest advancements and trends in chemistry, analysis, pharmacology and pharmacokinetics. *Current Organic Chemistry*.

[54] Guo L, Bai S-P, Zhao L, Wang X-H (2012). Astragalus polysaccharide injection integrated with vinorelbine and cisplatin for patients with advanced non-small cell lung cancer: effects on quality of life and survival. *Medical Oncology*.

[55] Zhuge Z-Y, Zhu Y-H, Liu P-Q (2012). Effects of astragalus polysaccharide on immune responses of porcine PBMC stimulated with PRRSV or CSFV. *PLoS ONE*.

[56] Du XG, Zhao B, Li JY (2012). Astragalus polysaccharides enhance immune responses of HBV DNA vaccination via promoting the dendritic cell maturation and suppressing Treg frequency in mice. *International Immunopharmacology*.

[57] Guo LW, Liu JG, Hu YL (2012). Astragalus polysaccharide and sulfated epimedium polysaccharide synergistically resist the immunosuppression. *Carbohydrate Polymers*.

[58] Schulz KF, Altman DG, Moher D (2010). CONSORT 2010 statement: updated guidelines for reporting parallel group randomised trials. *Journal of Clinical Epidemiology*.

